# High Prevalence and Genotypic Diversity of the Human Papillomavirus in Amazonian Women, Brazil

**DOI:** 10.1155/2013/514859

**Published:** 2013-08-13

**Authors:** Danielle Albuquerque Pires Rocha, Roberto Alexandre Alves Barbosa Filho, Francisca Andrade de Queiroz, Cristina Maria Borborema dos Santos

**Affiliations:** ^1^Universidade Federal do Amazonas, Estrada Coari-Mamiá 305, 69460-000 Coari, AM, Brazil; ^2^Alfredo da Matta Foundation, Rua Codajás 24, Cachoeirinha, 69065-130 Manaus, AM, Brazil

## Abstract

The aim of this study was to determine the prevalence of human papillomavirus (HPV) in a women population living within the state of Amazonas, Brazil, and to determine the viral genotypes found. The study included 361 sexually active women over 18 years of age. We performed the Pap test and the molecular diagnosis for HPV DNA using polymerase chain reaction (PCR). The amplicons obtained were sequenced in automatic sequencer for genotyping. The presence of HPV DNA was found in 29.1% (105) of the women. Only 321 women presented satisfactory slides for cytological diagnosis, 97.9% (314) had normal cytology (negative for cancer), and 2.1% (7) had abnormal cytology (4 ASCUS, 1 LSIL, and 2 HSIL). The types more frequently found were HPV 16 (58.1%) and HPV 58 (20.0%). Additionally, we found more 13 types of HPV. Compared with previous studies in Brazil, our data confirmed a high prevalence and genotypic diversity of HPV in Brazilian women.

## 1. Introduction

Cervical cancer, whose most strongly associated etiologic agent is the HPV, a sexually transmitted pathogen, is a leading cause of death among women [1]. In 2013, the National Cancer Institute estimates that there will be approximately 17,540 new cases of this disease in Brazil, occupying the third position among the most common cancers affecting among women, second only skin cancer (not melanoma) and breast cancer. For the state of Amazonas, the numbers are even more concerning because it is estimated that this disease will occupy the first position among the most common cancers in women [2].

There are over 100 genotypes of HPV, identified by numbers indicating the sequence of historical description. They are divided into groups according to oncogenic risk: low risk (e.g., HPV6 and HPV11, related to the development of benign lesions) and high risk (mainly represented by HPV16 and HPV18, involved in malign transformation of epithelium) [3, 4]. Reports of the prevalence of HPV demonstrate that the infection of HPV 16 is the most prevalent in the world. However, the frequency of the other types of HPV exhibits a wide geographical variability [5, 6].

The Amazon region is known as an area with a high level of sexually transmitted infections (STI) and high incidence of cervical cancer. It is a region that has a large territory, where there are many isolated communities in rural and indigenous areas where access is possible only by river, and even small urban areas have a very precarious health system, making hard the access to primary and secondary preventions. The aim of this study was to determine the prevalence of infection for HPV in a population of women living within the state of Amazon and to determine the viral genotypes found.

## 2. Methods

### 2.1. Population and Sample Collection

This is a prevalence study, conducted in the city of Coari, in the state of Amazonas, Brazil. Coari is a small town with an urban population of about 68 000 inhabitants, whose access can be done by river and air. It was a typical city in the Amazon until a few years ago, but with the discovery and exploitation of petroleum in the city, there was the arrival of workers coming from various other regions of Brazil in the past ten years. In this context, changes in sexual behavior in this population tend to happen, especially among women. Considering the prevalence of infection of HPV in a previous Brazilian study of 56.0% [7], with margin of error of 5% and trust of 95%, the sample consisted of 361 women, users of health care services in that city, sexually active, from 18 to 78 years old (mean 36.4 years old, SD = 13.4). The participants were interviewed, and they provided informed consent. They were examined by nursing staff, and cervical samples were obtained for Pap test and PCR for detection of HPV DNA. The cytology specimens were classified as normal (negative for malignancy), reactive changes, squamous atypia of indeterminate significance (ASCUS), low-grade squamous intraepithelial lesions (LSIL), high-grade squamous intraepithelial lesions (HSIL), and invasive carcinoma.

### 2.2. DNA Extraction

400 *μ*L of proteolytic TPK buffer (TE [Tris-HCl 50 mM + EDTA 50 mM, pH = 8.0], Tween 20% e proteinase K 10 mg/mL) was added to 400 *μ*L of each sample, and the samples were incubated for 60 minutes at 56°C and for 10 minutes at 95°C in dry bath. Then, DNA was extracted for phenol/chloroform method [8], precipitated with absolute ethanol, and resuspended in 50 *μ*L of ultrapure water (pH = 7.6). Quantification of DNA was performed in equipment NanoDrop to check the extraction efficiency.

### 2.3. PCR for HPV DNA Detection

Nested PCR was performed for HPV DNA detection. The first reaction was performed using the primers MY09 and MY11 [9], which amplify a fragment with 450 bp. The reaction final volume was 25 *μ*L, containing 5 U of Platinum Taq DNA Polymerase (Invitrogen, Brazil), 5 pmoL of each primer, 2.5 *μ*L of reaction buffer 10X, 50 mM of MgCl_2_, 10 mM of dNTP, 2.5 *μ*L of sample, and water. The second reaction was performed using the primers GP5+ and GP6+ [10], which amplify a fragment with 150 bp. The reaction final volume was 25 *μ*L, containing 5 U of Platinum Taq DNA Polymerase (Invitrogen, Brazil), 5 pmoL of each primer, 2.5 *μ*L of reaction buffer 10X, 50 mM of MgCl_2_, 10 mM of dNTP, 0.5 *μ*L of sample, and water. Both reactions obeyed the following thermocycler: 95°C for 1 minute, 40 cycles of 95°C for 1 minute, 55°C for 1 minute, and 72°C for 1 minute, ending with 72°C for 5 minutes.

In the reactions of this study, water was included as a negative control and a sample previously tested as positive control. The reactions were performed in a thermocycler Veriti (Applied Biosystems). The amplification products were subjected to electrophoresis on agarose gel 2.0% stained with ethidium bromide (1 *μ*g/*μ*L) and then visualized with the aid of a transilluminator, and the image was captured by digital camera Olympus SP-500uz.

### 2.4. HPV Genotyping by Gene Sequence

All HPV-positive samples were purified with alkaline phosphatase and Exonuclease (ExoSap System, GE Healthcare). The sequencing reaction was performed in a specific plate, in the thermocycler Veriti (Applied Biosystems), with final volume of 10 *μ*L, containing 0.3 *μ*L of Big Dye (Applied Biosystems), 2 *μ*L of buffer 5X, 5 pmoL of primer (MY09, MY11, GP5+, or GP6+), 2 *μ*L of sample, and water. The reaction times and temperatures were 96°C for 1 minute, 15 cycles of 96°C for 10 seconds, 50°C for 15 seconds, 60°C for 75 seconds, followed by 5 cycles of 96°C for 1 minute, 15 cycles of 96°C for 10 seconds, 50°C for 15 seconds, 60°C for 90 seconds, followed by 5 cycles of 96°C for 1 minute, 15 cycles of 96°C for 10 seconds, 50°C for 15 seconds, and 60°C for 120 seconds. The precipitation of the sequencing plate was accomplished, and then the sequences were sequenced on ABI 3130XL automated sequencer (Applied Biosystems) according to manufacturer's instructions. For genotyping, the files generated by the automated sequencer were compared with the sequences deposited in GenBank.

### 2.5. Data Analysis

The data were analyzed using the software Epi Info 3.5.3. For the quantitative variables, the mean and standard deviation were calculated when the data presented normal distribution, and the non-parametric test of Mann-Whitney was applied in the rejection of the normality hypothesis. For the categorical variables, the simple and relative absolute frequencies were calculated and, in some cases, the trust interval was at a level of 95%. Still in the analysis of categorical data, the chi-square test of Pearson or the exact test of Fisher was calculated. The significance level fixed in the application of the tests was 5%.

### 2.6. Ethics

This research was conducted in compliance with all ethical issues listed to research with human beings, and the research project was approved in Ethical Research Committee of the Federal University of Amazonas.

## 3. Results

The prevalence of infection for HPV in the studied women was 29.1% (*n* = 105, IC 95% 24.5–34.1). The types of HPV more frequently found were HPV 16 (58.1%) and HPV 58 (20.0%). Additionally, more 13 types of HPV (33, 81, 6, 70, 31, 35, 45, 52, 53, 61, 68, 71, and 89) were found. On physical examination, there were no changes indicative of HPV infection, such as warts or condyloma in any participant.

Of the 361 participant women, only 321 presented satisfactory slides for cytological diagnosis (slides considered unsatisfactory for cytological examination showed a large amount of blood and/or thick layer of cells), 314 of them (97.9%) presented normal cytology, and 7 (2.1%) presented cytological alterations (4 ASCUS, 1 LSIL, and 2 HSIL). Of the women without cytological alterations, 94 (29.2%) presented infections by HPV, and among the 7 who presented cytological abnormalities, 5 (71.4%) were infected. Among the women with normal cytological exam, we found the prevalence of 31.8% of infection with HPV 16.  Table 1 shows the types of HPV found in women with altered cytology.

The infected women presented a mean of age of 36 years old (SD = 13.2). When we analyzed the age groups, we found that the largest prevalence in the age group was between 55 and 64 years old, followed by the age group from 25 to 34 years old (31.3%). In relation to the educational degree, we found in the infected group a prevalence a little larger of women with complete high school in relation to those with incomplete high school, but the difference was not statically significant (*P* = 0.322). A smaller prevalence of infections was found in women with larger number of sexual partners (above 10) than women who had from 1 to 5 and from 6 to 10, without, however, presenting a statically significant difference (*P* = 0.119). About the age of the first sexual intercourse, the means of the ages were very close, being 15.7 (SD = 2.4) years old for the HPV positive women and 15.9 (SD = 2.5) for the HPV-negative women (*P* = 0.32).

Other risk factors related to the sexual behavior were analyzed (marital status, use of condom with fixed or eventual sexual partners, previous history of STI, and relationship with partner with previous history of STI), without, however, showing significant differences of prevalence, and when they showed, there were not statically significant differences among the groups infected and non infected.

## 4. Discussion

Although the state of Amazonas is an area with a high prevalence of cervical cancer [2], studies about the infection of HPV in Amazonian women are scarce. In this research, the prevalence of infection with HPV was high in the women studied. Almost 30% of them were infected, and of these, almost 60% were infected with HPV 16, a high-risk HPV. Studies conducted in other Brazilian cities, accomplished in women during gynecological routine exam, showed variable prevalence, but almost always high: 21.0% in Florianópolis [11], 56.0% and 35.3% in Rio de Janeiro [7, 12], and 35.0% in women from São Paulo [13].

It is very important to establish the types of circulating HPV in different regions, considering the differences related to the clinical endings that the high-risk and low-risk types carry. This knowledge is important also in trying to foresee the impact of the anti-HPV vaccines existent in the market, whether they would be really effective or not in a certain population.

In this study, we found a high prevalence of HPV 16 in infected women. This virus type is considered a high-risk oncogenic type, and together with HPV 18, is considered the viral type most often implicated in the development of cervical cancer worldwide [4, 6, 14, 15]. This result is concerning, since women infected with high-risk oncogenic types have higher rates of progression to cervical intraepithelial neoplasia compared with those infected with low-risk oncogenic viruses. In another study conducted in the state of Amazonas both with and without cytological abnormalities, HPV 16 was also the most frequently encountered [16].

We emphasize the fact that, in our sample, we did not find any women positive for HPV 18. A not very striking presence of HPV 18 can be a feature found in this region of the country; in a research conducted by Castro et al. (2011) [16] in the city of Manaus, the authors did not find any case of HPV 18, and the HPV 16 was also predominant (56.0%). Accordingly, studies in other Brazilian regions have also found low prevalence of HPV 18, both in women without cytological abnormalities and in women with precursor lesions and cancer [5, 17].

We have to considerer, however, the intrinsic characteristic of the genotyping method used in this research (gene sequencing) of not being able to detect coinfections but just the virus type present in greater quantity. Hybridization using type-specific probes and (restriction fragment length polymorphism) RFLP can detect coinfections. However, gene sequencing is the only one able to detect all HPV types and variants present in biological specimens [18].

HPV 58 was significantly found in this study. This type is closely related to HPV 16, both belonging to nine specie of the alpha-papillomavirus genus [3, 6]. Worldwide studies bring HPV 58 as the seventh most common type with precursor lesions and cervical cancer and the sixth most common type in women without cytological abnormalities, but differences in prevalence rates have shown marked regional differences. The highest prevalence of HPV 58 is in Latin America and Asia. Studies have found high prevalence of this type in China, Japan, Mexico, Costa Rica, Colombia, and Brazil [4–6, 19, 20].

The remaining 21.9% of HPV genotype found exhibited some varieties. Excluding the most prevalent types (HPV 16 and 58), 13 other types of HPV were found, all of low risk (81, 6, 61, 70, 71, and 89), probably high risk (53 and 68), and high-risk (33, 31, 35, 45, and 52), according to Muñoz et al. (2006) [21]. Other Brazilian studies have found greater genotype variety [14, 17, 22].

Of the 7 women with abnormal cytology, five were infected with HPV, all high-risk HPV (16, 33, and 58). Just one patient with ASCUS did not exhibit infection. This is plausible, because this classification is related to the presence of reparative morphological alterations, instead of no-neoplastic, therefore being most reversible. According to Burd (2003) [23], approximately 30%–50% of the cases of ASCUS are related to HPV. He affirms that one of the advantages of the combination of molecular detection of HPV with cytology is to assist the diagnosis of women who had indeterminate results in cervical screening. A study by Musa et al. (2010) [24] found a higher prevalence because 43.2% with ASCUS were positive for HPV infection.

One patient with HSIL had no HPV infection. The literature shows some studies with similar findings. Magalhães et al. (2008) [7], studying 42 women with HSIL, found 14 (33.3%) negative for HPV. According to them, some possible explanations for a negative result of HPV by a molecular method as opposite to positive cytology are low cellularity and occurrence of false negative result, since the method does not have 100% efficacy [7].

Among patients with normal cytology, we found prevalence of 31.8% of HPV infection, which can be considered high. Worldwide studies show prevalence between 9.6% and 51.0% of infections for HPV in healthy women [4, 25, 26], with remarkable geographic diversity. In our study, this high prevalence of HPV-positive women but without cytological abnormalities is significant because although HPV infection can be transient, persistent maintenance of the infection can put all these women in a group at risk for developing cervical cancer, and therefore, those women should be monitored more carefully.

The prevalence of HPV infection is higher among young women, with peak infection in women under 25 years of age, declining with advancing age. This pattern of infection is possibly due to the development of adaptive immune response by the infected individuals, preventing future infection [27]. In this study, infected women had a mean age of 36.0 years (SD = 13.2). We emphasize, however, that only women over the age of 18 were included in this study, which contributed to the fact that the mean age was not lower. Additionally, when we analyzed age groups, we found a higher prevalence in the age group between 55 and 64 years (34.6%), followed by the age group 25–34 years (31.3%). This phenomenon, a second peak of infection after the age of 55, has been seen in different parts of the world [27, 28]. The reason for this second peak is still unclear. It may be due to reactivation of latent infections, due to gradual loss of type-specific immunity, or changes in the pattern of sexual behavior in the last two decades, with the acquisition of new infections [11].

These results taken together demonstrate the existence of a “silent epidemic” of HPV infection in women belonging to this population, and therefore efforts must be made to reduce the incidence of this infection and its associated diseases, especially cancer of the cervix uterus. The molecular diagnosis and genotyping of this virus have been widely employed in epidemiological studies worldwide. The implementation of this method in routine diagnosis in difficult places, like much of the Amazon region, however, presents difficulties such as the high cost and the need for adequate infrastructure and qualified staff. The monitoring by cytology and HPV vaccination is shown as the best strategy to decrease the high mortality rates of cervical cancer in this state.

The HPV vaccination is not a part of the Brazilian immunization schedule. However, some cities have implemented the vaccine in their populations. The state government of Amazonas recently announced that it will implement in the coming years the vaccine for all adolescents. Although we did not find cases of HPV 11 and HPV 18 among the infected women, the stinking HPV 16 infection in this population suggests that the anti-HPV vaccine available would be of great impact in reducing the infection in this population. However, genotypic diversity found in Latin American and Asian countries, in particular the striking HPV 58 infection in these populations, points to the need to build more regionalized vaccines.

## 5. Conclusion

In this study we found a high prevalence of HPV infection in Amazonian women who were seen in heath centers for routine pelvic examination. Despite the considerable genotype variety, HPV 16 was the most prevalent, affecting 58.1% of infected women. This high prevalence of HPV 16 suggests that the anti-HPV vaccine, the bivalent or the tetravalent one, could have a high impact on reducing the incidence of cervical cancer in this region, although the variety of other circulating types is still a cause of concern. Other studies about the distribution of HPV types worldwide are necessary to direct future vaccine development according to regional needs.

## Figures and Tables

**Table 1:** Presence or absence in women with altered cytology and viral types found in infected women in the city of Coari, Amazonas, Brazil.

Cytology exam	Presence/absence of HPV	Viral type
ASCUS	Presence	16
ASCUS	Presence	16
ASCUS	Presence	58
ASCUS	Absence	—
LSIL	Presence	33
HSIL	Absence	—
HSIL	Presence	16

ASCUS: squamous atypia of undetermined significance; LSIL: low-grade squamous intraepithelial lesions; HSIL: high-grade squamous lesions.
